# Supplementary material for: Amplitude of circadian rhythms becomes weaken in the north, but there is no cline in the period of rhythm in a beetle
Source: PLoS One. 2021 Jan 14;16(1):e0245115. doi: 10.1371/journal.pone.0245115 (PMC7808652; doi:10.1371/journal.pone.0245115)
Supplement: Attachment — Submitted filename: Responses to Reviewers 20201112.docx [file pone.0245115.s003.docx]

12th November 2020

Dear Reviewers:

'Response to Reviewers'

Thank you for allowing us to revise and so much for your many useful suggestions for our paper “PONE-D-20-27518, Amplitude of circadian rhythms becomes weaker in the north, but there is no cline in the period of rhythm in a beetle”. We have revised the manuscript as per the referee requests and hope the revised version is now suitable for publication in *'*' PLOS ONE”. We have addressed all of the reviewer’s comments – see our responses below. The author responses to the reviewer’s comments are as follows. Each reviewer’s comment is highlighted in bold, and our responses follow immediately below.

For the reviewer #1

**Reviewer #1: This is an interesting ms concerning the geographical variability of the circadian rhythm phenotype in T castaneum. The literature is mixed on this subject with classical Pittendrigh experiments in Drosophila species in Japan suggesting that the amplitude of the rhythm is stronger at higher latitudes because it has to counterbalance the effects of extreme photoperiods which would tend to make animals arrhythmic. There is not much molecular evidence to back this up, indeed recent studies from Helfrich-Foerster suggest the opposite, in that a weaker circadian rhythm in northern latitudes is more flexible for adaptation of circadian behavior to extreme photoperiodic conditions. Costa's group suggests that the circadian light response in D. melanogaster should be reduced at higher latitudes - this pair of papers (Science, 2007???) is not cited.**

Author responses: Thank you for highlighting that our manuscript has potential and for your interesting thoughts on our manuscript. We have followed your suggestions and revised the manuscript following your recommendations. We are appreciate it for your teaching the two important papers which show similar results from our study. We altered the phrase (Lines 69-75) with the references (Lines 376-382).

**Discuss the Helfrich-Foerster and Costa studies on weaker fly circadian rhythms in northern latitudes mentioned by reviewer 1.**

Author responses: Thank you for your teaching the two important papers which show opposite results from our study. We have cited these, and discussed with these studies (Lines 185-211).

**Consequently it would have been nice if the study had also included a phase shift response to light as that would have been perhaps more relevant at these latitudes. Nevertheless, this study comes out in favour of the latter hypothesis. The results reflect a lot of work and the ms is clear to read and understand.**

Author responses: Thank you for your useful suggestion. Unfortunately, we are sorry to inform you that it is not possible to conduct this experiment with these wild beetle strains because most of these local populations are not currently being kept in captivity. We're sorry we can't fix it here, but we're very glad you found this study interesting.

**My one quibble is that the effects are tiny and require a huge N to be observed. Also the variation in latitude is only 8oC which doesn't help to dissect out an effect. Furthermore the power statistic used is not very sophisticated and there are better methods available, but I suspect the result would be the same.**

Author responses: Thank you for your suggestion. We can't come up with a better way to improve on the status quo, and we think that using other methods would not change the results as you suggested, so we didn't alter this point. Instead, in lines 269-278, we added a discussion of the too large sample size and weak detection effect in this analysis.

**The Discussion is a bit too long considering the brevity and conciseness of the results, so perhaps a little less speculation would be in order. Otherwise, the study is OK.**

Author responses: We have reduced the amount of consideration according to your and another reviewer's points. Also we removed some phrase to short the discussion of the revised manuscript.

Thank you for your useful comments, and we hope the revised manuscript is suitable for publication.

With best wishes,

Takahisa Miyatake

On behalf of all authors

---------------------

For the reviewer #2

**Reviewer #2: The authors conducted a study on the effects of geographic (primarily latitudinal) variation on circadian rhythmicity in the red flour beetle. Strengths of the study included the large sample size and rearing the populations for 2 generations in the lab to separate out maternal/environmental effects. While the English was professionally edited, I often found the ideas to be communicated in an imprecise or unclear manner.**

Thank you for highlighting that our manuscript has potential and for your interesting thoughts on our manuscript. We have followed your suggestions and revised the manuscript following your recommendations.

**1. It was completely unclear why the authors repeatedly referred to longitude early in the manuscript (e.g., L29, L40...a key word!, L47, L53, L78). The study is primarily designed to detect latitudinal clines given the much larger latitudinal range sampled. The authors state that longitudinal clines are also known (L47) but don't give a citation. Based on their titles, all the references cited on L54 (refs 8-12) refer to latitudinal clines. I can understand why they would want to test for this possibility within their data, but I don't know that there is any a priori reason to expect a longitudinal cline. This was also very confusing to me because they referred to altitudinal clines (L26 of abstract) making me think that maybe these would be investigated, but think they were ever discussed in the manuscript. A third point of confusion is that the authors said they were analyzing effects of temperature (L130), but I don't think they ever did.**

Author responses: Thank you for your suggestions. “Altitude” in L.26 is my writing miss, and thus I changed it to “longitude”, instead of “altitude”. Sorry for “effects of temperature is also my miss writing, and thus we removed “temperatures” and then altered it to “To analyze the effects if latitude and sex on the period and power of…”. (L 138-139).

**2. The discussion on L61-69 is very unclear. The authors seem to be mixing points related to "where flies live" and latitude. By "where flies live" do they mean geographic features that might be distinct from latitude? If so what are they? L67 "pioneering"? Some of the studies cited here are quite old ("pioneering"). Is there a consensus emerging in the newer literature? The discussion overall was hard to follow.**

Author responses: We changed the wording that is taken to be geographic distribution to latitude, and we've added a couple of recent papers to the list (Lines 66-75).

**3. The predominant finding of the paper was that there is an effect of latitude on amplitude/power of the circadian rhythm. This finding would be more useful if it were put into some kind of context. What is the seasonal variation in photoperiod at the northern vs. southern end of the range? What are the typical temperature ranges? How does this compare with other studies in which circadian clines were or weren't found?**

Author responses: Added the context in discussion as you requested (Lines 190-211).

**4. While the sample size was apparently quite large, it was described in a very confusing way. For example, L109-110 makes it sound like only one beetle was measured per population. Maybe one was measured per population in each trial and several were monitored at once? L117/TableS1 is confusing because it sounds like it would be the number of beetles measured per population, but I think this is the starting population that was collected and used for breeding (??).**

Author responses: Sorry for the confusion caused by our writing style. The sample size of Table S1 shows individual numbers measured for circadian rhythm. Therefore, the number of individuals with measured circadian rhythms is really very, very large. Therefore, we added the phrase “We measured the circadian rhythms of 17 to 128 individual beetles for a single population”, in lines 116-118, and we placed the following phrase “The sample size measured for circadian rhythm of each population is shown in Table S1”, just after the previous sentence.

**5. L113 the mention of "a clear Petri dish" again confused me...these are the same petri dishes referred to in L110, right? How many petri dishes were monitored at a time? Was there one animal in each dish? I was really confused.**

Author responses: At a time, we monitored 128 individuals; each animal was put it in each Petri dish. We have prepared 128 locomotor activity sensors. Thus we added the phrase “At a time, we monitored 128 individuals, meaning we are measuring 128 sensors at the same time”, in Lines 123-124.

**6. L151-152. This description was inadequate. In what direction was the relationship, and how did it compare with the latitudinal effect on amplitude/power? I think this is important because total activity may affect the power to detect a change in amplitude or period (if animals aren't moving much it's harder to detect a change). Are these trends strong enough that such an indirect effect might be important?**

Author responses: Thank you for your helpful comments. We have improved the sentence about the relationship (Lines145-146).

Since there was a weak negative correlation between activity and power of rhythm (Spearman’s correlation coefficient = -0.22), lower activities do not necessarily cause lower rhythm.

Moreover, we conducted path analysis to detect the direct and indirect effects. We found the significant direct effect of latitude on power of rhythm and the similar estimated regression coefficient (-1.84 +- 0.76(SE)) to the one reported in the main text (-2.35 +- 1.08(SE)) although there was the significant indirect effect (latitude -> activity -> power of rhythm).

Therefore, we concluded that higher latitude caused lower rhythm although the effect size was small. Likewise, the result of the period was qualitatively same with the one in the main text.

Altered the phrase to “The results of the regression analysis showed that total activity was associated positively with latitude and sex (Table 1, Figure 4).”

**7. L158 this statement is really vague. Are the authors talking about clines or just inter-individual variation? What are they trying to say?**

Author responses: Removed it.

**8. L166-167 The transition to this sentence is really rough. "On the other hand" would usually indicate a contrast with the previous statement, which isn't the case here.**

Author responses: Removed it.

**9. L176-177 This sentence seemed like an odd way to end the paragraph. And it is a sort of contrast to the opening sentence (L168-9). Why not combine them into one strong sentence at the beginning. Apparently, a limited number of studies have identified this trend. It's fine to just say that (once) and would be even better if the authors could put it in context. Are there just a few because it's only occasionally studied? Because most studies don't have sufficient power, or because it seems to be rare?**

Author responses: We combined the two phrases, as you requested.

**10. L188-194 This section again convoluted. L188-190 is a wordy sentence that should be streamlined/clarified. The examples should be clearly organized to indicate which support wide dispersal and which don't. My sense is that both Drury (33) and Arnold (34) suggest limited dispersal. This is obscured by the use of "on the other hand" which implies a contrast. On top of this the text about Semeao's study is repeated twice with very similar text (L195-197 and L233-237).**

Author responses: We've put together a short series of these sentences as you requested, and removed the sentence from Semeao's study, which appears a second time, to avoid sentence duplication, as you requested (Lines 240-242).

**11. L252 I would argue that not only do they need molecular studies of the circadian rhythmicity (e.g., clines in alleles of specific circadian genes), but also to couple genetic studies (i.e. population assignment) more directly with phenotypic studies.**

Author responses: Altered the last phrase as you requested.

**Minor
L51 "indicators of circadian rhythms" is vague. These are characteristics of rhythms, and variations in these characteristics could indicate adaptation etc.**

Author responses: Altered as you requested (Lines　50-51).

**L55 "Additionally" here doesn't make sense. The topic sentence makes it sound like they are switching to talking about "insects other than Drosophila" but the preceding paragraph is also mostly about insects and there is no mention of Drosophila.**

Author responses: Altered as you requested (Lines　54).

**L58-59 a positive relationship between "latitude and circadian rhythm" doesn't make sense. I think the authors might mean a positive relationship between latitude and period.**

Author responses: Altered as you requested (Lines 57-58).

**L75 The syntax is hard to follow. I suggest "This species is found throughout most of Japan, except for the northern island of Hokkaido, meaning..."**

Author responses: Changed it as you requested (Lines 81-83).

**L89-91 Please write the coordinates in a more standard way.**

Author responses: Rewrote (Lines 95 and 96).

**L101-103 These two sentences almost completely repeat each other.**

Author responses: Removed the latter sentence (see Lines 106-107).

**L106 "To assess circadian rhythmicity" or "To assess characteristics of circadian rhythms" or "To assess circadian phenotypes"**

Author responses: Changed to “"To assess circadian rhythmicity" (Line 110).

**L123 periodogram is misspelled.**

Author responses: I think “periodogram” is not misspelled. Instead, altered from “periodgram” to “periodogram” in Lines 129 and 131.

**L137 power of the rhythm**

Author responses: Altered as you requested (Line 139).

**L138-139 this description was confusing. It made it sound like they were only considering one (presumably latitude), but in fact they considered each of them in separate analyses.**

Author responses: Explained precisely more for this point (Lines 138-139).

**L218 "if without human cleaning" doesn't make any sense. I kind of understand that humans cleaning out the pests would disrupt the colonization/selection/evolution, but that's not explained.**

Author responses: Thank you for your suggestion, and thus we altered the phrase to “including human cleaning which may affect to colonization, selection and evolution of creatures in each mill” (Lines 240-242).

**L236 I might misunderstand, but wouldn't lack of isolation suggest higher gene flow?**

Author responses: There was an error in our original manuscript. We altered it as the correct description “the isolation at each mill suggested lower levels of gene flow…” (Lines 258-259).

**L242 suggest "is not occurring on the spatial scale that we examined"**

Author responses: Changed as you requested (Lines 264-265).

**L246 significantly smaller than what? I think the authors just mean significant p values.**

Author responses: Altered the phrase to “Notably, the results of the present study showed P-values smaller than 0.05, even for small effect sizes, due to the very large sample size” in Lines 269-270.

**L253 I don't know what the authors mean by different phenotypic phenomena than other insect species.**

Author responses: Removed it.

**Table 1 was misaligned in my version.**

Author responses: Rewrote Table 1, and added “Adjusted R-squared”, an index to show the effect size as the editor requested.

Thank you for your useful comments, and we hope the revised manuscript is suitable for publication.

With best wishes,

Takahisa Miyatake

On behalf of all authors
