# Supplementary material for: Amplitude of circadian rhythms becomes weaken in the north, but there is no cline in the period of rhythm in a beetle
Source: PLoS One. 2021 Jan 14;16(1):e0245115. doi: 10.1371/journal.pone.0245115 (PMC7808652; doi:10.1371/journal.pone.0245115)
Supplement: Attachment — Submitted filename: Responses to Reviewers.docx [file pone.0245115.s004.docx]

18th December 2020

Dear Dr. Henrik Oster (Academic Editor),

Thank you for allowing us again to revise and so much for your many useful suggestions for our paper “PONE-D-20-27518R1, Amplitude of circadian rhythms becomes weaken in the north, but there is no cline in the period of rhythm in a beetle”, and for the two expert reviews. We have revised the manuscript as per the referee requests and hope the revised version is now suitable for publication in '' PLOS ONE”. We have addressed all of the reviewer’s comments – see our responses below.

PONE-D-20-27518R1

Amplitude of circadian rhythms becomes weaker in the north, but there is no cline in the period of rhythm in a beetle

PLOS ONE

The author responses to the reviewer’s comments are as follows. Each reviewer’s comment is highlighted in bold, and our responses follow immediately below.

**==============================**

**Please fix all minor issues raised by the reviewers.**

- We fixed all issued raised by the reviewers.

**Further, it is highly recommended to get support from a native speaker or a professional editing service to improve the English of the manuscript.**

- The English of the re-revised manuscript has been checked by a professional native English editing service.

==============================

**6. Review Comments to the Author

Please use the space provided to explain your answers to the questions above. You may also include additional comments for the author, including concerns about dual publication, research ethics, or publication ethics. (Please upload your review as an attachment if it exceeds 20,000 characters)**

**Reviewer #1: The ms is interesting but the English really needs to be improved before publication so some editorial assistance is required. Also the organisation of the Discussion could be better. Here are a few further comments.**

- We are glad that you are interested in this paper. The English of the re-revised manuscript has been checked by a professional native English editing service.

**L66-68. Incorrect statement. The long TIM isoform, not the short one, is negatively correlated with latitude ie higher frequency at lower latitudes.**

- Sorry for our mistake. We corrected it as you requested (Lines 66-69).

**L203-205 Again the timeless statement is not correct.
‘The frequency of a clock gene ( timeless ) of D. melanogaster studied from 40 to 65 degrees in latitude was negatively phased with latitude, but in populations col lected in southern
Europe by other years , this negative trend seemed to be unclear [21] .’
If the author wishes to use the timeless story, which I think has an important relevant message for this ms. which the authors do touch upon in their discussion of local adaptations and bottlenecks later on, perhaps they might use the paragraph below. I suggest the timeless part be moved to the latter part of the discussion.
‘The frequency of the timeless allele encoding a long TIM isoform of D. melanogaster initially studied from 40 to 65oN was initially negatively correlated with latitude, but in populations collected in extreme southern Europe (32-35oN) this trend was reversed [21]. It turned out that the latitudinal cline was actually caused by natural selection spreading the recently derived tim allele in all directions from a point of origin in southern Italy, thereby generating a distance rather than a latitudinal cline. This ‘distance cline’ was confirmed by studying the tim allele in the Iberian Peninsula (reference Zonato et al 2018 PMID: 29183263) where the cline was reversed. These studies reveal that the history of the genes that encode clinal characteristics also need to be considered when attempting to interpret spatial distributions. Such historical perspectives may account for some of the contradictory results that have been generated in the geographical analyses of biological rhythm phenotypes.**

- Sorry for our mistake, again. We rephrased and moved it to the later part of the discussion as you requested (Lines 291-303) and added the reference (Lines 468-471).

**L221 the power of a rhythm is not necessarily the amplitude. It is the general sinusoidal shape of the rhythm. Thus a very sinusoidal but low amplitude rhythms could still have considerable power.**

- Removed “power” (Line 217)

**L229 ‘rear flying’????? explain**

- Reword it as “short-range flying” (Line 225)

**L291-293. Suggest ‘ Furthermore, given the seasonal differences in light exposure at different latitudes, studying the phase shift of the locomotor rhythm to brief light pulses might also provide a relevant phenotype that might discriminate between northern and southern population, as was the case for Drosophila timeless variants (21)**

- Thank you so much for your suggestion for the last phrase. We rephrased it as you requested (Lines 285-289).

**Reviewer #2: L25 missing word "these traits are" (?)**

- Added “are” (Line 25).

**L55-59 It takes a little "mental gymnastics" for a reader to compare a trend in "higher latitudes" with "a positive relationship with latitude". I suggest adding something to indicate that the two studies are showing trends in the same direction. Like "Similarly, a positive relationship..."**

- Added “Similarly,“ (Line 57).

**L66 I'm not quite sure what is meant by "frequency of a clock gene". I think the authors mean something like period/frequency of expression. Also not quite sure what is meant by "negatively phased" (negatively correlated?)**

- Altered to “The frequency of the timeless allele encoding a long TIM isoform,” (Lines 66-67), and to “negatively correlated” (Line 68-69), as you requested. Thank you for your suggestions.

**L69 I'm not really sure what's meant by "The other context of circadian rhythm". It's not really clear how this is really different from the previous paragraphs where the authors were also talking about relationships with latitude and rhythms. Seasonal variation is mentioned but not really explained.**

- We removed the phrase (Line 69).

**L71 pioneering**

- Re-worded (Line 70).

**L74 wording doesn't seem right, and it seems trivial to say that the results will be discussed in the context of the literature. Perhaps eliminate this sentence, especially since the next sentence acknowledges the discrepancies.**

- Eliminated the sentence (Lines 73-74).

**L82 The authors know more about this than I do, but I usually see this name written as "Hokkaido" in English. Confirm that the spelling is as desired.**

- You are quite right. (Line 81).

**L101 this sentence seems to repeat the sentence starting on L91.**

- Removed the sentence (see Line 100).

**L107 Suggest deleting this sentence as it is essentially repeated in the sentence beginning on L109. The sentence beginning on L109 seems better because the association with grain is the part most similar to natural conditions. The temperature and photoperiod presumably vary among sites.**

- Removed the sentence (see Line 106).

**L152-154 I don't understand what the authors are saying here. If you are trying to analyze the relationship between two things (geographical area and power) you need to include them in the same model (?) I think they perhaps mean that effects of latitude and sex were analyzed separately? Or maybe they mean 3 things: latitude, longitude and sex?**

- Sorry to confuse you. The two things were latitude and longitude. Therefore, we rewrote this phrase as “To avoid multicollinearity between latitude and longitude (the correlation coefficient (r) between them was 0.83), we didn’t include both simultaneously in the model as an explanatory variable but constructed a separate model with each value.” (Lines 149-152).

**L198-199 this is misleading sounds like it spans a range of 32 degrees. Suggest something like "in a range of approximately 8 degrees latitude (from about 32 to 41°N).**

- Altered it as you suggested (Lines 196-197). Thank you.

**L199 Since the authors make it a point that there are no effects of longitude, the longitudinal range (about 10 degrees) should also be given.**

- We gave it in Line 201.

**L199 The authors say "Previous studies have often" but only cite one study. Preferably cite several examples or refer to the reference in a different way like "(e.g., [21])" (to indicate that it is an example) or ([21] and references therein) if you are citing the reference as a sort of review.**

- Rephrased it as “([21] and references therein)” (Line 197-198).

**L203 see my comments about similar text within the introduction**

- Rewrote and moved it to the last of the discussion as you and the reviewer 1 commented (Lines 291-303).

**L206 what does "this negative trend seemed to be unclear" mean? A negative trend wasn't observed? Or maybe not significant?**

- We have corrected this sentence as noted by reviewer 1. (Lines 197-201).

**L207 it is possible that a cline could be detected**

- Added “a” (Line 203).

**L211 I don't really understand what the authors mean by "survey with closely related species". Do they mean extend the geographic range by pooling results from two species? This idea is unclear.**

- We rewrote it as “it may be possible to survey the point if we use a closely related species, such as *Tribolium confusum* who inhabits also in Hokkaido.” Is this make sense? If not the case, we will remove the phrase at the next revision. (Lines 206-208).

**L211 "In examining the clock cline between different species" is awkward wording. Maybe "In comparing clock clines among speices"**

- Changed it as you requested. (Line 208).

**L225-227 wording unclear...kind of repeats "suggest that this beetle disperses very well [35] suggested very high levels of active dispersal" (??) I think the second part of the sentence is meant to be an example. Maybe authors mean "....very well, e.g., Ridley et al. [35] inferred very high levels of active dispersal..."**

- Altered as you requested. (Line 221-222).

**L229 I don't know what "including rear flying" means**

- Altered to “short-range flying” (Line 225).

**L232; as follows**

- Changed (Line 228).

**L248 I'm not sure what the authors mean by ideal environment. I think they mean "ideal" for large effects of bottlenecks and local adaptation. It's not written clearly.**

- Reworded (Lines 244-245).

**L272 suggest "a cline in circadian amplitude"**

- Added “circadian” as you suggested (Line 267).

**L277-279 and L282-286 These two sentences say the same thing. Can they be consolidated?**

- Thank you for your advice. The two sentences have slightly different meanings and are difficult to concatenate. I hope you understand it (See 274-281).

**L288 This line doesn't make sense.**

- Reworded it (Line 283).

**L469 indicate threshold (p-value) for significance.**

- Added p-values in the captions of Figures 2 (*p*<0.05) and 4 (*p*<0.01), for lines 482-491, respectively.

Thank you for all of your kind suggestions and advices. Red text, other than where the reviewers have noted, is where a professional native English editing service has made corrections. We hope the revised version of the manuscript is now suitable for publication.

With best wishes,
Takahisa Miyatake

Behalf of all authors

Masato S. Abe, Kentarou Matsumura, Taishi Yoshii
